# Efficacy and safety of lumasiran for infants and young children with primary hyperoxaluria type 1: 12-month analysis of the phase 3 ILLUMINATE-B trial

**DOI:** 10.1007/s00467-022-05684-1

**Published:** 2022-08-01

**Authors:** Wesley Hayes, David J. Sas, Daniella Magen, Hadas Shasha-Lavsky, Mini Michael, Anne-Laure Sellier-Leclerc, Julien Hogan, Taylor Ngo, Marianne T. Sweetser, John M. Gansner, Tracy L. McGregor, Yaacov Frishberg

**Affiliations:** 1 Department of Paediatric Nephrology, Great Ormond Street Hospital, London, UK; 2 Division of Pediatric Nephrology and Hypertension, Mayo Clinic, Rochester, MN USA; 3 Pediatric Nephrology Institute, Rambam Health Care Campus, Haifa, Israel; 4 Paediatric Nephrology Unit, Galilee Medical Center, Nahariya, Israel; 5 Division of Nephrology, Department of Pediatrics, Texas Children’s Hospital, Baylor College of Medicine, Houston, TX USA; 6 Hôpital Femme Mère Enfant and Centre d’Investigation Clinique Inserm, Hospices Civils de Lyon, ERKnet, Bron, France; 7 Pediatric Nephrology Department, Hopital Robert-Debré, APHP, Paris, France; 8 Alnylam Pharmaceuticals, Cambridge, MA USA; 9 Division of Pediatric Nephrology, Shaare Zedek Medical Center and Faculty of Medicine, Hebrew University of Jerusalem, Jerusalem, Israel

**Keywords:** Hyperoxaluria, Primary, Infant, Nephrocalcinosis, RNA interference, Lumasiran

## Abstract

**Background:**

Primary hyperoxaluria type 1 (PH1) is a rare genetic disease that causes progressive kidney damage and systemic oxalosis due to hepatic overproduction of oxalate. Lumasiran demonstrated efficacy and safety in the 6-month primary analysis period of the phase 3, multinational, open-label, single-arm ILLUMINATE-B study of infants and children < 6 years old with PH1 (ClinicalTrials.gov: NCT03905694 (4/1/2019); EudraCT: 2018–004,014-17 (10/12/2018)). Outcomes in the ILLUMINATE-B extension period (EP) for patients who completed ≥ 12 months on study are reported here.

**Methods:**

Of the 18 patients enrolled in the 6-month primary analysis period, all entered the EP and completed ≥ 6 additional months of lumasiran treatment (median (range) duration of total exposure, 17.8 (12.7–20.5) months).

**Results:**

Lumasiran treatment was previously reported to reduce spot urinary oxalate:creatinine ratio by 72% at month 6, which was maintained at 72% at month 12; mean month 12 reductions in prespecified weight subgroups were 89%, 68%, and 71% for patients weighing < 10 kg, 10 to < 20 kg, and ≥ 20 kg, respectively. The mean reduction from baseline in plasma oxalate level was reported to be 32% at month 6, and this improved to 47% at month 12. Additional improvements were also seen in nephrocalcinosis grade, and kidney stone event rates remained low. The most common lumasiran-related adverse events were mild, transient injection-site reactions (3 patients (17%)).

**Conclusions:**

Lumasiran treatment provided sustained reductions in urinary and plasma oxalate through month 12 across all weight subgroups, with an acceptable safety profile, in infants and young children with PH1.

**Graphical abstract:**

A higher resolution version of the Graphical abstract is available as Supplementary information

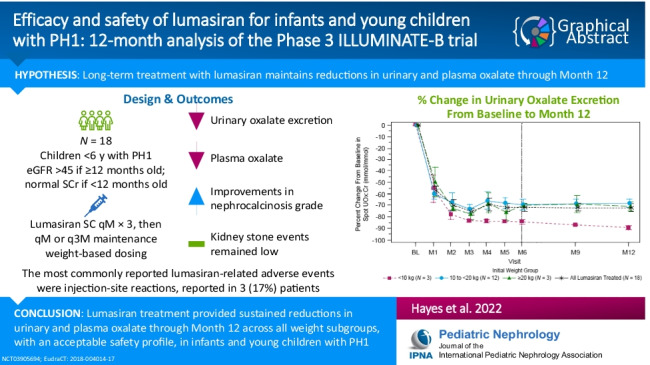

**Supplementary Information:**

The online version contains supplementary material available at 10.1007/s00467-022-05684-1.

## Introduction

Primary hyperoxaluria type 1 (PH1) is a rare, autosomal recessive disease characterized by hepatic overproduction of oxalate due to a deficiency in the peroxisomal enzyme alanine–glyoxylate aminotransferase (AGT), which is encoded by the *AGXT* gene [1, 2]. AGT catalyzes the conversion of glyoxylate to glycine in hepatic peroxisomes. When AGT is dysfunctional, glyoxylate is not effectively converted into glycine but instead undergoes oxidation to oxalate in the liver. Oxalate cannot be metabolized further and is eliminated primarily by the kidneys [3].

Excess oxalate combines with calcium to form insoluble calcium oxalate crystals in the kidney parenchyma and urinary tract, leading to nephrocalcinosis, recurrent kidney stones, and progressive kidney damage [1, 4]. As kidney function declines, the elimination of oxalate is reduced, resulting in an increase in plasma oxalate levels. Systemic oxalosis occurs when calcium oxalate crystals deposit in multiple organs, leading to bone, cutaneous, vascular, cardiac, ophthalmologic, and neurologic manifestations [1, 2].

Although PH1 affects patients of all ages and has a heterogeneous presentation [1], the onset of symptoms, most commonly nephrocalcinosis and recurrent kidney stones, occurs at a young age during childhood or adolescence in the majority of patients [4, 5]. Recurrent kidney stones and their associated hospitalizations and removal procedures are a major cause of morbidity in patients with PH1 [5]. Development of nephrocalcinosis is associated with the risk of kidney failure [6], and patients with untreated PH1 typically progress to kidney failure [5, 7]. Death from kidney failure and/or life-threatening complications of systemic oxalosis can occur [1, 8]. Patients who present with PH1 during infancy often have infantile oxalosis, characterized by the development of kidney failure before the age of 1 year and a high mortality rate [9–12].

Treatment options for patients with relatively preserved kidney function include hyperhydration and administration of crystallization inhibitors to reduce calcium oxalate crystallization, as well as high-dose pyridoxine (vitamin B6, a coenzyme for AGT) to improve AGT function and enable the normal metabolic pathway [1, 7, 13, 14]. After the disease has progressed to advanced chronic kidney disease (CKD), invasive approaches such as dialysis, liver transplantation, or combined liver/kidney transplantation become essential; the only definitive cure for PH1 is liver transplantation [1]. Even intensive dialysis may not be adequate to prevent systemic oxalosis, and liver transplantation carries a significant risk of morbidity and mortality [1, 15, 16].

Lumasiran is a liver-directed RNA interference (RNAi) therapeutic recently approved by the US Food and Drug Administration for the treatment of PH1 to reduce urinary oxalate levels in pediatric and adult patients [17] and by the European Commission for the treatment of PH1 in all age groups [18]. Lumasiran specifically targets and promotes degradation of the mRNA of glycolate oxidase (GO), which is encoded by *HAO1*. By inhibiting the production of GO, lumasiran decreases levels of glyoxylate, the immediate precursor of oxalate, thereby reducing hepatic oxalate production, while increasing concentrations of a readily excreted precursor, glycolate [17–19]. Lumasiran targets the upstream GO enzyme, resulting in substrate reduction, a mechanism of action independent of the underlying *AGXT* gene variant [17–19].

Although the potential application of RNAi therapies is increasingly recognized across rare disorders and in common conditions [20, 21], lumasiran is the first RNAi therapeutic approved for use in infants and young children [17, 22]. In the phase 3, multinational, open-label, single-arm ILLUMINATE-B study of 18 infants and children < 6 years old with PH1 and an estimated glomerular filtration rate (eGFR) > 45 mL/min/1.73 m^2^, lumasiran resulted in a substantial reduction in urinary oxalate excretion, as measured by spot urinary oxalate:creatinine ratio (UOx:Cr) (primary endpoint), from baseline to month 6 (averaged months 3–6; least-squares (LS) mean, 72.0%; 95% CI, 66.4–77.5%) [22]. Positive results were also demonstrated for all secondary endpoints evaluated in the 6-month primary analysis period [22]. Lumasiran had an acceptable safety profile during the primary analysis period; all adverse events (AEs) were mild to moderate with the most common lumasiran-related AEs being mild, transient injection-site reactions [22].

All active patients in ILLUMINATE-B (*N* = 18) entered the extension period (EP) at month 6 of the study. Here, we report interim safety and efficacy data from patients after they have completed at least 12 months on study in ILLUMINATE-B.

## Methods

### Study design and patients

ILLUMINATE-B is an ongoing 60-month study consisting of a 6-month primary analysis period followed by a 54-month EP. The study design and eligibility criteria for ILLUMINATE-B (ClinicalTrials.gov: NCT03905694; EudraCT: 2018–004014-17) have been described previously [22]. Briefly, patients < 6 years old with genetically confirmed PH1 and eGFR > 45 mL/min/1.73 m^2^ (if ≥ 12 months old) or normal serum creatinine (if < 12 months old) were eligible. For all patients, the dose of lumasiran was administered subcutaneously based on a body weight based dosing regimen, with dose adjustments for interval weight gain (Table S1).

### Efficacy endpoints

The primary endpoint was the percent change in urinary oxalate from baseline to month 6, assessed by UOx:Cr levels from random spot urine collections. To account for variability, 3 single-void samples were collected within 7 days prior to dosing, and the mean of the 3 samples was used for baseline and post-baseline assessments for all patients. Additional details are described in Sas et al. [22]. Secondary endpoints assessed in the EP included percent and absolute change from baseline in urinary oxalate excretion; proportion of patients with urinary oxalate excretion less than or equal to the upper limit of normal (≤ ULN) and ≤ 1.5 × ULN; percent and absolute change from baseline in plasma oxalate; lumasiran plasma pharmacokinetic parameters; and change from baseline in eGFR. Select exploratory endpoints included changes from baseline in nephrocalcinosis grade, kidney stone event rate, plasma glycolate, and the frequency of anti-drug antibodies (ADAs).

### Safety endpoints

Safety assessments included monitoring of AEs, clinical laboratory assessments, vital signs, 12-lead electrocardiography, and physical examination. Adverse events were coded according to the Medical Dictionary for Regulatory Activities version 23.0.

### Assessments

During the EP, maintenance dosing continued per the weight-based regimens (Table S1) and study site visits occurred at least every 3 months. Urine and blood samples were collected for measurement of oxalate and glycolate with validated liquid chromatography-tandem mass spectrometry (LC–MS/MS) assays at a central laboratory. The LC–MS/MS assays were developed by Alnylam to meet regulatory requirements and have not yet been published, but values for plasma oxalate and glycolate in healthy adults are generally higher than with other assays. Blood samples were also collected for lumasiran pharmacokinetic assessments. eGFR was calculated for patients ≥ 12 months old using the Schwartz Bedside formula [23]. Patients < 12 months old were excluded from the eGFR analysis, as the Schwartz Bedside formula is not validated for that age group.

Nephrocalcinosis grade was assessed at baseline, month 6, and month 12 by kidney ultrasound scans. The degree of medullary nephrocalcinosis per kidney was graded by a radiologist with central reads using a validated, semi-quantitative scale of 0 to 3, with a higher grade indicating greater severity [24]. Changes in nephrocalcinosis grade were grouped into 4 categories of overall change, accounting for both kidneys: no change, improving, worsening, and indeterminate (1 kidney improving and 1 worsening).

Kidney stone events were adjudicated by the investigator and included at least 1 of the following: visit to a healthcare provider (e.g., outpatient clinic, urgent care, emergency department, procedure) because of a kidney stone; medication for renal colic; stone passage; or macroscopic hematuria due to a kidney stone. The kidney stone event rate was calculated as the total number of kidney stone events divided by the total patient exposure time (events per person-year).

Anti-drug antibodies (immunoglobulin G, immunoglobulin M) against lumasiran were evaluated in plasma at baseline and months 1, 3, 6, 9, and 12 using a validated enzyme-linked immunoassay.

### Statistical analysis

This interim analysis was conducted using data at the cutoff date of February 3, 2021, after all active study patients had completed their month 12 visit. Secondary efficacy endpoints for the EP included percent change in urinary oxalate excretion from baseline as measured by spot UOx:Cr. Changes in plasma oxalate were also evaluated in the plasma oxalate analysis set, defined as all patients who had a baseline plasma oxalate level ≥ 1.5 times the lower limit of quantitation (LLOQ; 5.55 μmol/L). Values below the LLOQ were assigned a value of 5.55 μmol/L.

Safety analyses were conducted in all patients who received any amount of lumasiran; cumulative safety data from the first dose of lumasiran through the data cut-off (February 3, 2021) are reported. Pharmacokinetic parameters were evaluated in all patients who received at least 1 full dose of lumasiran and had at least 1 evaluable post-dose blood sample for pharmacokinetic assessment.

All study endpoints were summarized with descriptive statistics. For absolute and percent change in urinary and plasma oxalate, the mean with corresponding standard error of the mean was calculated. Prespecified subgroup analyses by initial weight-based dosing subgroups were performed to further understand the treatment effect.

## Results

### Patients

Of the 18 patients enrolled in the 6-month primary analysis period, all entered the EP and completed at least an additional 6 months of treatment with lumasiran. The median (range) total duration of lumasiran exposure was 17.8 (12.7–20.5) months. Patients received 7 to 19 doses of lumasiran depending on weight, with a cumulative total of 173 doses. No patient missed any dose. A total of 18, 16, and 9 patients were treated with lumasiran for ≥ 12, ≥ 15, and ≥ 18 months, respectively. Baseline patient demographics and clinical characteristics were reported previously [22] and are summarized in Table 1.

**Table 1:** Baseline demographic and clinical characteristics of the patients

	< 10 kg (*N* = 3)	10 to < 20 kg (*N* = 12)	≥ 20 kg (*N* = 3)	All treated (*N* = 18)
Age at consent, median (range), months	10.1 (3–14)	50.1 (23–72)	62.2 (54–72)	50.1 (3–72)
Age at diagnosis, median, months	0.8	22.7	27.0	16.3
Time from diagnosis to first dose date, median, months	11.6	28.6	46.4	23.5
Genotype^a^, *n* (%)				
PR/*	0	3 (25)	0	3 (17)
M/M or M/N	1 (33)	8 (67)	1 (33)	10 (56)
N/N	2 (67)	1 (8)	2 (67)	5 (28)
Pyridoxine use, *n* (%)	2 (67)	7 (58)	2 (67)	11 (61)
Spot UOx:Cr, median (range), mmol/mmol^b^	1.253 (1.126‒1.708)	0.453 (0.166‒1.205)	0.350 (0.255‒0.693)	0.469 (0.166‒1.708)
Plasma oxalate, median (range), μmol/L^c^	22.3 (17.2‒30.6)	9.6 (6.6‒19.9)	11.7 (7.2‒18.7)	11.5 (6.6‒30.6)
In plasma oxalate analysis set^d^	22.3 (17.2‒30.6)	11.8 (8.7‒19.9)	15.2 (11.7‒18.7)	13.7 (8.7‒30.6)
eGFR, median (range), mL/min/1.73 m^2e^	135 (135‒135)	111 (76‒174)	90 (65‒135)	111 (65‒174)
History of kidney stone events in past 12 months, *n* (%)	0	2 (17)	1 (33)	3 (17)
Presence of nephrocalcinosis at baseline, *n* (%)	3 (100)	10 (83)	1 (33)	14 (78)

*eGFR* estimated glomerular filtration rate, *LLOQ* lower limit of quantitation, *UOx:Cr* urinary oxalate:creatinine ratio

^a^M = missense; N = nonsense; PR = pyridoxine-responsive; * = any genotype of PR, M, or N. PR was defined as NM_000030.3(AGXT):c.508G > A (p.Gly170Arg) or NM_000030.3(AGXT):c.454T > A (p.Phe152Ile). M and N were defined based on a publication by Mandrile et al. [26]

^b^1 mmol/mmol = 0.796 mg/mg; 1 mmol/mmol = 1000 mmol/mol

^c^Upper limit of normal = 12.11 μmol/L for plasma oxalate, as determined based on data from 75 healthy adults

^d^In patients with baseline plasma oxalate ≥ 1.5 × LLOQ (5.55 μmol/L; *N* (all treated) = 13)

^e^eGFR (mL/min/1.73 m^2^) was calculated based on the Schwartz Bedside formula [23] for patients ≥ 12 months; *N* (all treated) = 16. eGFR was not calculated for 2 patients because their age at baseline was < 12 months

### Efficacy

#### Urinary oxalate

**Fig. 1:**
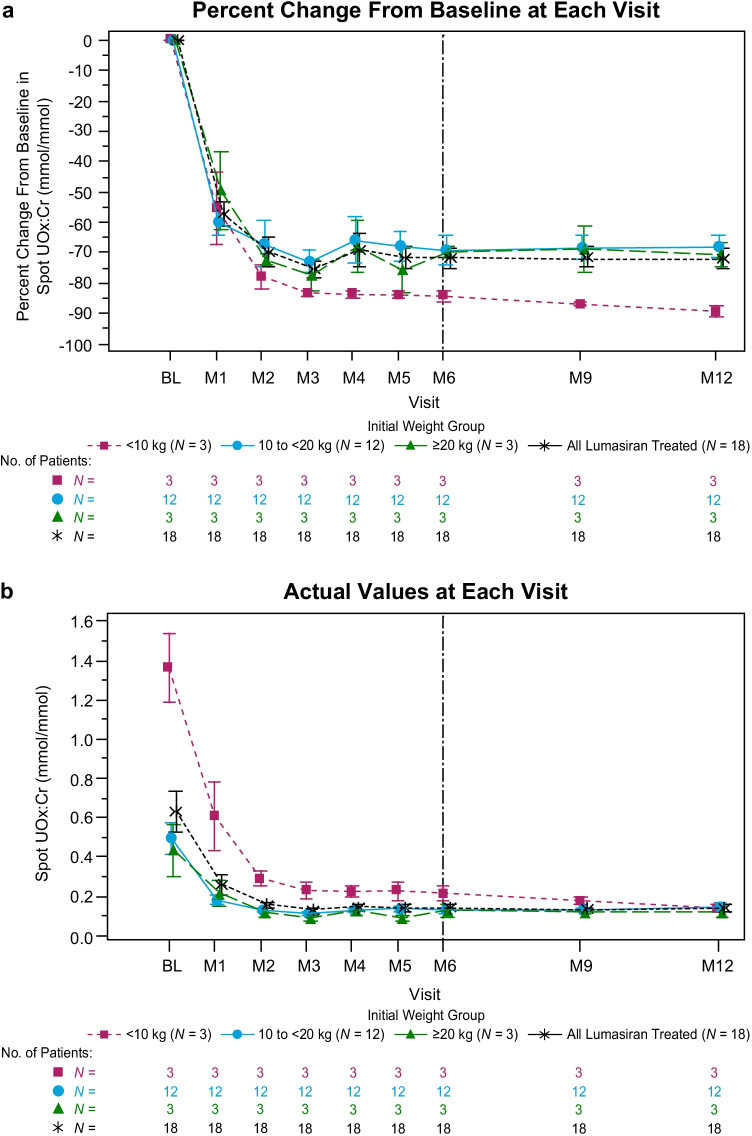
Mean (SEM) spot UOx:Cr. **a** Percent change from baseline at each visit and **b** actual values at each visit. Baseline value was the mean of all assessments collected prior to the first dose of lumasiran; 1 mmol/mmol = 0.796 mg/mg; 1 mmol/mmol = 1000 mmol/mol. BL, baseline; M, month; UOx:Cr, urinary oxalate:creatinine ratio

**Fig. 2:**
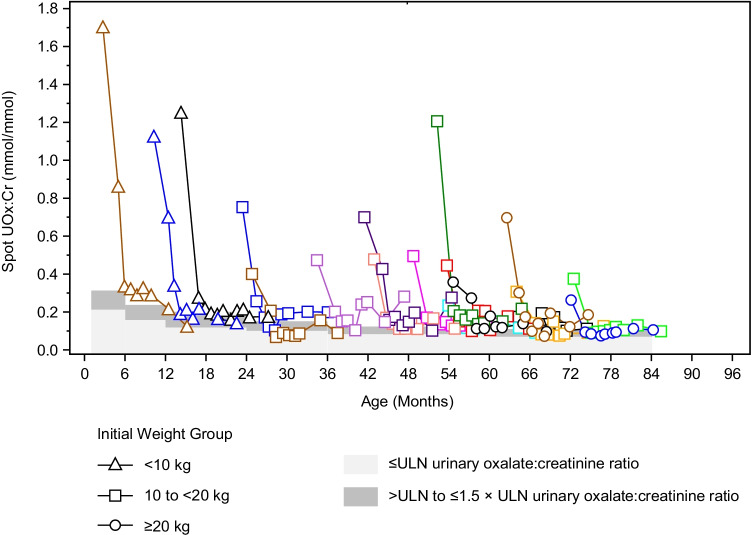
Actual spot UOx:Cr values by age; 1 mmol/mmol = 0.796 mg/mg; 1 mmol/mmol = 1000 mmol/mol. ULN, upper limit of normal; UOx:Cr, urinary oxalate:creatinine ratio

Long-term treatment with lumasiran led to sustained reductions in urinary oxalate excretion, as measured by spot UOx:Cr (Figs. 1 and 2). The rapid and sustained reduction in spot UOx:Cr from baseline to month 6 was maintained to month 12 across all weight subgroups (Fig. 1; Fig. S1). The mean (SEM) reduction from baseline was 72% (3.4%) or 0.49 (0.09) mmol/mmol at month 6 and 72% (3.2%) or 0.49 (0.1) mmol/mmol at month 12 (Table 2; Fig. 1). All patients showed a substantial reduction in spot UOx:Cr from their baseline levels to month 12, regardless of age (Fig. 2). The ratio of spot UOx:Cr to age-dependent ULN, which accounts for the age-related decline in urinary oxalate in infants and young children [25], showed similar results: the mean (SEM) percent reduction from baseline was 69% (4.0%) at month 6 and 69% (3.5%) at month 12 (Fig. S1). At month 6 and month 12, 9 and 10 of 18 patients achieved near normalization (≤ 1.5 × ULN) of spot UOx:Cr, respectively, including 1 patient at month 6 and 2 patients at month 12 who achieved normalization (≤ ULN) (Table 2). Similar sustained reductions were observed in the 24-h urinary oxalate analysis in patients who were able to provide a sample (*N* = 2 at month 6; *N* = 4 at month 12) (Table 2).

**Table 2:** Secondary efficacy endpoints

	Lumasiran (*N* = 18)	
	Month 6	Month 12
Percent change from baseline in spot UOx:Cr, mean (SEM)	− 71.7 (3.4)	− 71.9 (3.2)
Absolute change from baseline in spot UOx:Cr, mean (SEM), mmol/mmol^**a**^	− 0.49 (0.09)	− 0.49 (0.10)
Patients with spot UOx:Cr, *n* (%)		
≤ ULN^b^	1 (6)	2 (11)
≤ 1.5 × ULN^b^	9 (50)	10 (56)
Percent change from baseline corrected for body surface area in 24-h urinary oxalate, mean (SEM), mmol/24 h/1.73 m^2c^	− 68.41 (5.60)	− 63.23 (7.23)
Absolute change from baseline corrected for body surface area in 24-h urinary oxalate, mean (SEM), mmol/24 h/1.73 m^2c^	− 1.35 (0.08)	− 1.24 (0.29)
Percent change from baseline in plasma oxalate, mean (SEM)^d^	− 32.06 (6.71)	− 47.14 (4.59)
In plasma oxalate analysis set, mean (SEM)^d,e^	− 37.40 (8.83)	− 56.43 (3.85)
Absolute change from baseline in plasma oxalate, mean (SEM), μmol/L^d^	− 5.0 (1.3)	− 7.3 (1.5)
In plasma oxalate analysis set, mean (SEM)^d,e^	− 6.5 (1.6)	− 9.5 (1.7)
Change from baseline in eGFR, mean (SEM), mL/min/1.73 m^2f^	− 0.3 (3.9)	− 1.5 (4.4)
Percent change from baseline in eGFR, mean (SEM)^f^	0.9 (3.0)	− 0.9 (4.1)

*eGFR* estimated glomerular filtration rate, *LLOQ* lower limit of quantitation, *ULN* upper limit of normal, *UOx:Cr* urinary oxalate:creatinine ratio

^a^One mmol/mmol = 0.796 mg/mg; 1 mmol/mmol = 1000 mmol/mol

^b^Age-dependent ULN [25]

^c^In patients with valid 24-h urinary oxalate measurements; *N* = 2 at month 6 and *N* = 4 at month 12

^d^ULN = 12.11 μmol/L for plasma oxalate, as determined based on data from healthy adults

^e^In patients with baseline plasma oxalate ≥ 1.5 × LLOQ (5.55 μmol/L [*N* = 13]; values below LLOQ were assigned a value of 5.55 μmol/L)

^f^eGFR (mL/min/1.73 m^2^) was calculated based on the Schwartz Bedside formula [23] for patients ≥ 12 months; *N* = 16 at month 6

#### Plasma oxalate

**Fig. 3:**
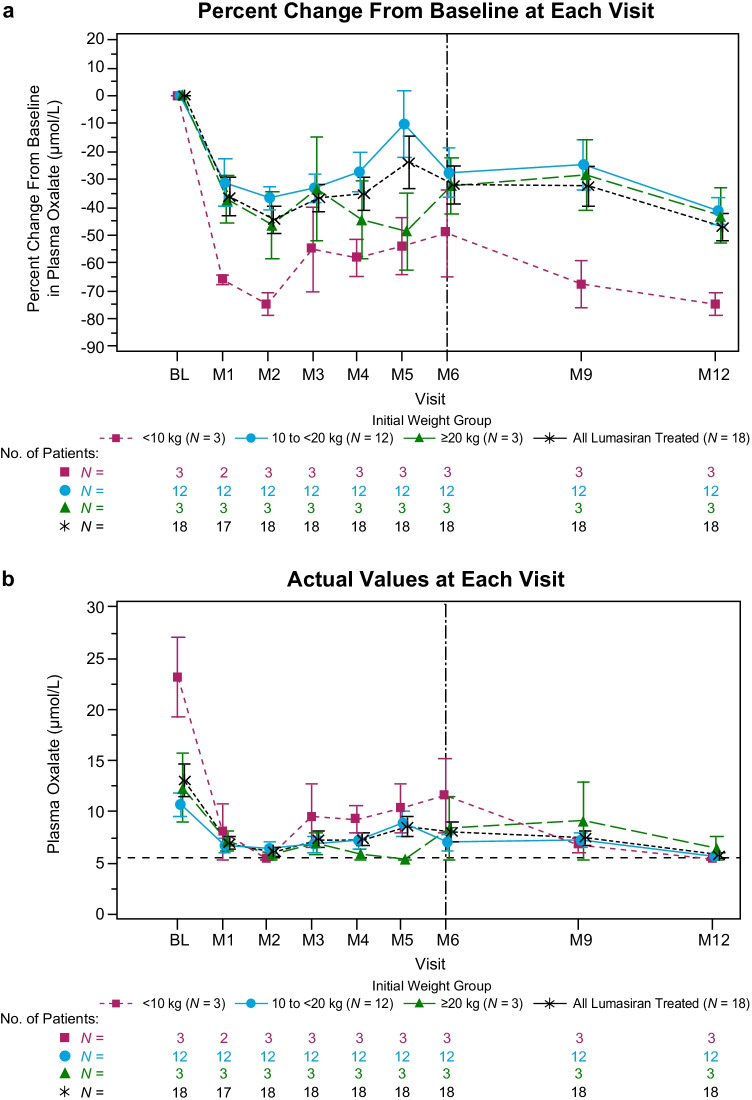
Mean (SEM) plasma oxalate. **a** Percent change from baseline and **b** actual values at each visit. Baseline value was the mean of all assessments collected prior to the first dose of lumasiran. Dotted line in **b** represents lower limit of quantitation. Values below this threshold were assigned a value of 5.55 µmol/L. BL, baseline; M, month

Plasma oxalate levels were reduced after 6 months of lumasiran treatment, and the reduction was sustained at month 12 across all weight subgroups (Fig. 3). The mean (SEM) plasma oxalate levels at baseline, month 6, and month 12 were 13.24 (1.53) µmol/L, 8.21 (0.94) µmol/L, and 5.91 (0.19) µmol/L, respectively. The mean (SEM) reductions in plasma oxalate levels from baseline to month 6 and month 12 were 32% (7%) or 5.03 (1.29) µmol/L, and 47% (5%) or 7.33 (1.49) µmol/L, respectively (Fig. 3; Table 2). In patients with baseline plasma oxalate levels ≥ 1.5 × LLOQ (*N* = 13), the mean (SEM) reduction was 37% (9%) or 6.46 (1.63) µmol/L from baseline to month 6 and 56% (4%) or 9.51 (1.71) µmol/L from baseline to month 12 (Table 2).

#### Plasma pharmacokinetics and eGFR

Plasma pharmacokinetic parameters were generally similar across dosing weight subgroups and consistent with those reported in the primary analysis period [22] (Table S2). There is no accumulation of lumasiran with repeated dosing; therefore, plasma pharmacokinetic parameters are not expected to change over time. eGFR remained stable through month 12 (Table 2; Fig. S2), and serum creatinine remained stable through month 12 in the 2 patients < 12 months old at baseline.

#### Nephrocalcinosis

**Fig. 4:**
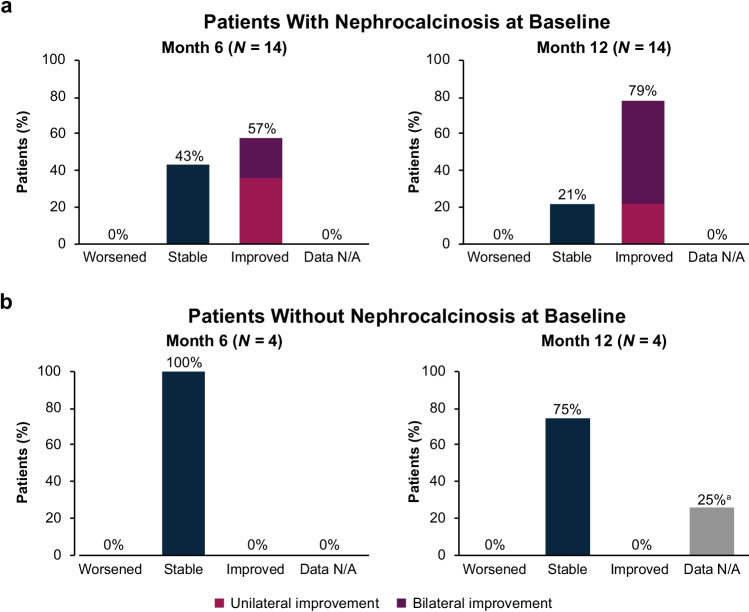
Change from baseline at month 6 and month 12 in nephrocalcinosis grade for patients with nephrocalcinosis at baseline **a** and without nephrocalcinosis at baseline **b**. ^a^Indeterminate (one side improves and the other side worsens) is not graphed because there were 0 cases. ^b^Unable to show improvement as patients did not have nephrocalcinosis at baseline. ^c^Data not available for one patient in the ≥ 20 kg weight subgroup who did not have kidney ultrasound at month 12. N/A, not available

Nephrocalcinosis was present at baseline in 14/18 (78%) patients. Of these 14 patients, nephrocalcinosis grade improved in 57% (8/14) after 6 months of lumasiran treatment. The proportion of patients with improvement increased to 79% (11/14) after 12 months of lumasiran treatment, and no patient worsened. At baseline, 4/18 (22%) patients did not have nephrocalcinosis (Table 1). After 6 months of lumasiran treatment, all 4 of these patients remained stable, with no worsening. After 12 months of treatment, of the 4 patients without nephrocalcinosis at baseline, 3 remained stable, and data were unavailable for 1 patient (Fig. 4).

#### Kidney stones

**Fig. 5:**
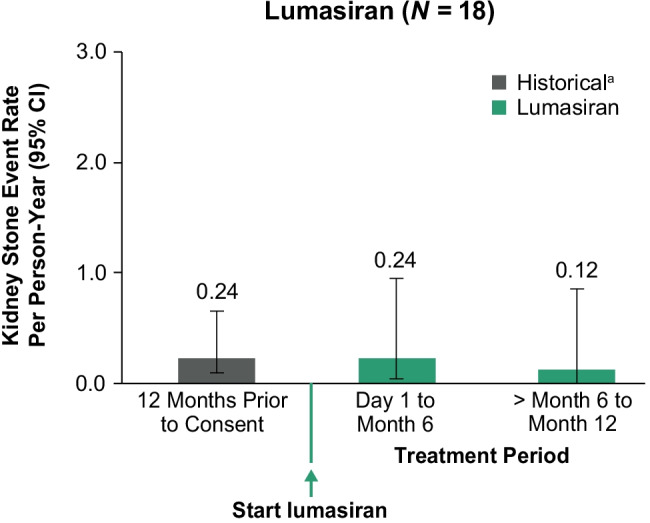
Kidney stone events. ^a^Historical group: patient-reported history of kidney stone events; annualized rate was not calculated for patients < 6 months old

The low rates of kidney stone events were unchanged from 12 months prior to consent through the first 6 months of lumasiran treatment and remained low during the additional 6 months of lumasiran treatment (Fig. 5).

#### Plasma and urinary glycolate

Plasma and urinary glycolate levels initially increased and then plateaued and remained stable from month 6 to month 12, consistent with a reduction in hepatic GO activity mediated by lumasiran (Fig. S3).

#### Immunogenicity

Transient, low titer (1:50) ADAs were observed in 3 of 18 (17%) patients during the 6-month primary analysis period, with no observable effect on efficacy, safety, or pharmacokinetics [22]. There were no new ADAs observed during the time period from month 6 to month 12.

### Safety

As of the data cutoff date (February 3, 2021), all 18 (100%) patients reported at least 1 AE (Table 3). All AEs were mild or moderate in severity; no severe AEs were reported. Adverse events reported in ≥ 3 patients overall were pyrexia, vomiting, rhinitis, upper respiratory tract infection, and injection-site reaction (Table 3). The most common AEs related to lumasiran were injection-site reactions, reported in 3 (17%) patients. All injection-site reactions were mild in severity and transient; symptoms included erythema, discoloration, and pain at the injection site. One of the 3 patients with injection-site reactions had mild, transient injection-site pain at the time of injection that resolved within 10 min.

**Table 3:** Safety

Event, *n* (%)	Patients			
	< 10 kg (*N* = 3)	10 to < 20 kg (*N* = 12)	≥ 20 kg (*N* = 3)	All treated (*N* = 18)
AEs	3 (100)	12 (100)	3 (100)	18 (100)
AEs occurring in ≥ 3 patients overall				
Pyrexia	2 (67)	4 (33)	2 (67)	8 (44)
Vomiting	1 (33)	2 (17)	2 (67)	5 (28)
Rhinitis	1 (33)	3 (25)	0	4 (22)
Upper respiratory tract infection	1 (33)	2 (17)	1 (33)	4 (22)
Injection-site reaction	0	2 (17)	1 (33)	3 (17)
AEs leading to discontinuation of study treatment	0	0	0	0
AEs leading to withdrawal from the trial	0	0	0	0
Death	0	0	0	0
Serious AEs	0	0	1 (33)^a^	1 (6)
Severe AEs	0	0	0	0

Safety data from first dose of lumasiran to data cutoff date of February 3, 2021

*AEs* adverse events

^a^One patient had a serious AE of viral infection (moderate in severity; considered unrelated to lumasiran by the investigator) during the 6-month primary analysis period, which has been reported previously [22]

One patient (6%) had a serious AE of viral infection (moderate in severity; considered unrelated to lumasiran by the investigator) during the 6-month primary analysis period, which has been reported previously [22], and no additional serious AEs were observed. No deaths occurred, and no AEs led to treatment discontinuation, treatment interruption, or withdrawal from the study. There were no clinically relevant changes in laboratory measures (including hematology, blood chemistries, liver function tests, and kidney parameters), vital signs, physical examinations, or electrocardiograms related to lumasiran.

## Discussion

Infants and young children with PH1 can progress rapidly to kidney failure and systemic oxalosis, resulting in high rates of mortality [9, 10], and precipitating events such as obstructive kidney stones or acute dehydration can hasten the progression to kidney failure [5]. Treatment options for PH1 have been limited to conservative measures and pyridoxine for patients with relatively preserved kidney function [14, 26] and invasive methods such as dialysis and combined liver/kidney transplantation for patients with advanced CKD [1]. Given that elevated levels of urinary oxalate play a causal role in the development of kidney stones, nephrocalcinosis, and kidney failure [1, 4], effectively reducing hepatic oxalate production is important to improve symptoms and slow disease progression. As kidney function declines, plasma oxalate levels increase, contributing to systemic oxalosis, which causes significant morbidity and mortality in these patients. Furthermore, elevated plasma oxalate may serve as a predictor of kidney function decline in PH1 patients with CKD stages 3b–5 [27], and development of nephrocalcinosis has been correlated with the risk of kidney failure in PH1 patients [6], highlighting the importance of sustained stabilization or improvement of these measures.

RNAi therapeutics, which have the ability to reduce the expression of a single gene, are particularly useful in genetic diseases such as PH1. ILLUMINATE-B is the first study to demonstrate the efficacy and safety of the liver-targeted RNAi therapeutic lumasiran in a population of infants and young children [22]. The results of the current analysis of ILLUMINATE-B show that with 12 or more months of treatment, lumasiran demonstrated sustained efficacy and safety, comparable with results reported during the 6-month primary analysis period [22]. Specifically, lumasiran treatment achieved sustained reductions in spot UOx:Cr and plasma oxalate levels through month 12, with a low rate of kidney stone events. In this cohort, plasma oxalate levels were increased at baseline, despite relatively preserved kidney function, and declined after lumasiran treatment. Long-term treatment with lumasiran appeared to preserve kidney function as demonstrated by stable eGFR and an increased proportion of patients with an improvement in nephrocalcinosis grade. While these 12-month results are favorable, it will be important to continue to follow up with these patients in the EP (up to 54 months).

Lumasiran demonstrated an acceptable safety profile in this population of young patients with PH1. All AEs were mild to moderate in severity, and AEs considered related to lumasiran treatment were mild, transient injection-site reactions and headache. No patient discontinued treatment or withdrew from the study.

Results of the present analysis align with those from the 12-month analysis of the ILLUMINATE-A trial (NCT03681184), an ongoing, randomized, double-blind, phase 3 trial of lumasiran carried out in individuals ≥ 6 years old with PH1 and relatively preserved kidney function. In that trial, long-term lumasiran treatment was efficacious, with sustained lowering of urinary and plasma oxalate [28] and encouraging results for exploratory measures of kidney stone event rates and nephrocalcinosis grade [29], and the safety profile was consistent with that observed in the ILLUMINATE-B trial.

This study has several limitations. First, it was a single-arm, open-label study without a placebo control group. However, lumasiran has previously demonstrated significant reductions from baseline versus placebo in urinary and plasma oxalate in the 6-month, double-blind, placebo-controlled phase of the ILLUMINATE-A trial [30]. Second, the current trial excluded patients with more advanced kidney disease. However, the ongoing single-arm, open-label, phase 3 ILLUMINATE-C trial (NCT04152200) is evaluating lumasiran in patients with PH1 with advanced kidney impairment. Third, spot urine samples were collected; 24-h urine samples were limited in this cohort of infants and young children. However, a correlation between spot UOx:Cr and 24-h urinary oxalate has been demonstrated (*r* = 0.352; 0.63; 0.75; 0.79) [31–34]. The support for using spot UOx:Cr levels has been discussed in detail in the primary study report [22]. Finally, the current follow-up duration in the EP is limited; longer-term follow-up data are needed to evaluate the clinical benefits of lumasiran, such as resolution of nephrocalcinosis and improvement in eGFR.

In conclusion, this analysis of the ongoing phase 3 ILLUMINATE-B study showed that 12 months of treatment with lumasiran resulted in sustained reductions in urinary and plasma oxalate levels across all weight ranges in infants and young children who enrolled at < 6 years of age with PH1, with an acceptable safety profile. Given the causal role of urinary oxalate in kidney damage, maintenance of stable kidney function, low rates of kidney stone events, and the observed trend in improvement of nephrocalcinosis grade during long-term lumasiran treatment are encouraging. These results provide an example of an RNAi-based therapeutic being utilized effectively with an acceptable safety profile in young patients with PH1.

## Supplementary Information

Below is the link to the electronic supplementary material.

Supplementary file1 (DOCX 521 KB)

Graphical Abstract (PPTX 99.5 KB)

## Data Availability

Because of the sensitive nature of the data collected for this study, the dataset will not be made available to other researchers. The redacted Study Protocol and Statistical Analysis Plan are included in the Supplement.
