# Supplementary material for: Efficacy and safety of lumasiran for infants and young children with primary hyperoxaluria type 1: 12-month analysis of the phase 3 ILLUMINATE-B trial
Source: Pediatr Nephrol. 2022 Aug 1;38(4):1075–86. doi: 10.1007/s00467-022-05684-1 (PMC9925547; doi:10.1007/s00467-022-05684-1)
Supplement: Supplementary file 1 — Supplementary file1 (DOCX 521 KB) [file 467_2022_5684_MOESM1_ESM.docx]

**Supplementary Material to:**

# Efficacy and Safety of Lumasiran for Infants and Young Children with Primary Hyperoxaluria Type 1: 12-month Analysis of the Phase 3 ILLUMINATE-B Trial

Wesley Hayes, MBBChir^1^; David J. Sas, DO^2^; Daniella Magen, MD^3^; Hadas Shasha-Lavsky, MD^4^; Mini Michael, MD^5^; Anne-Laure Sellier-Leclerc, MD^6^; Julien Hogan, MD, PhD^7^; Taylor Ngo, MPH^8*^; Marianne T. Sweetser, MD, PhD^8^; John M. Gansner, MD, PhD^8^; Tracy L. McGregor, MD^8*^; Yaacov Frishberg, MD^9^

**Tracy L. McGregor, MD, and Taylor Ngo, MPH, were employees of Alnylam Pharmaceuticals at the time of study.*

^1^Department of Paediatric Nephrology, Great Ormond Street Hospital, London, UK; ^2^Division of Pediatric Nephrology and Hypertension, Mayo Clinic, Rochester, MN, USA; ^3^Pediatric Nephrology Institute, Rambam Health Care Campus, Haifa, Israel; ^4^Paediatric Nephrology Unit, Galilee Medical Center, Nahariya, Israel; ^5^Division of Nephrology, Department of Pediatrics, Texas Children’s Hospital/Baylor College of Medicine, Houston, TX, USA; ^6^Hôpital Femme Mère Enfant and Centre d’Investigation Clinique Inserm, Hospices Civils de Lyon, ERKnet, Bron, France; ^7^Pediatric Nephrology Department, Hopital Robert-Debré, APHP, Paris, France; ^8^Alnylam Pharmaceuticals, Cambridge, MA, USA; ^9^Division of Pediatric Nephrology, Shaare Zedek Medical Center and Faculty of Medicine, Hebrew University of Jerusalem, Jerusalem, Israel

**Corresponding Author:**

Wesley Hayes, MBBChir, Consultant Paediatric Nephrologist

Department of Paediatric Nephrology

Great Ormond Street Hospital

Great Ormond Street, London WC1N 3JH

London, UK

Phone: +44 (0)20 7762 6822

Email: [Wesley.Hayes@gosh.nhs.uk](mailto:Wesley.Hayes@gosh.nhs.uk)

**Table S1.** Dosing Regimen

| Body Weight | Loading Dose | Maintenance Dose  (begin 1 month after the last loading dose) |
| --- | --- | --- |
| <10 kg | 6.0 mg/kg once monthly for 3 doses | 3.0 mg/kg once monthly |
| 10 kg to <20 kg | 6.0 mg/kg once monthly for 3 doses | 6.0 mg/kg once every 3 months (quarterly) |
| ≥20 kg | 3.0 mg/kg once monthly for 3 doses | 3.0 mg/kg once every 3 months (quarterly) |

**Table S2.** Plasma Pharmacokinetic Parameters^a^

|  | <10 kg | | 10 to <20 kg | | ≥20 kg | |
| --- | --- | --- | --- | --- | --- | --- |
|  | Day 1 | Month 12 | Day 1 | Month 12 | Day 1 | Month 12 |
| Parameter Statistic | 6.0 mg/kg | 3.0 mg/kg | 6.0 mg/kg | 6.0 mg/kg | 3.0 mg/kg | 3.0 mg/kg |
| **t_max_** |  |  |  |  |  |  |
| n | 3 | 3 | 12 | 12 | 3 | 2 |
| Median (min, max), hours | 4.22 (2, 8.1) | 2.25 (2, 4) | 2.97 (1.87, 7.83) | 3.83 (1.75, 7.25) | 3.93 (1.97, 7.1) | 4.15 (4.03, 4.27) |
| **C_max_** |  |  |  |  |  |  |
| n | 3 | 3 | 12 | 12 | 3 | 2 |
| Mean (%CV), ng/mL | 950 (32.2) | 1960 (80.1) | 1030 (33.6) | 1520 (55.5) | 950 (121.1) | 509 (19.0) |
| **AUC_0-last_** |  |  |  |  |  |  |
| n | 3 | 3 | 12 | 12 | 3 | 2 |
| Geometric mean (%CV), h•ng/mL | 6810 (19.7) | 7170 (126.5) | 9160 (21.2) | 10,500 (39.1) | 4940 (36.2) | 2960 (128.1) |
| **t_½_** |  |  |  |  |  |  |
| n | 1 | 2 | 10 | 5 | 2 | 1 |
| Geometric mean (%CV), hours | 5.46 | 2.12 (45.4) | 4.82 (46.4) | 3.81 (27.0) | 3.33 (181.8) | 6.64 |
| **CL/F/WT** |  |  |  |  |  |  |
| n | 1 | 2 | 10 | 5 | 2 | 1 |
| Geometric mean (%CV), L/h/kg | 0.908 | 0.459 (1.5) | 0.574 (18.8) | 0.461 (23.4) | 0.513 (33.6) | 0.444 |

AUC_0-last_, area under the plasma concentration versus time curve from 0 to the last measurable concentration; %CV, percent coefficient of variation; CL/F, apparent total clearance of the drug from plasma; C_max_, maximum plasma concentration; max, maximum; min, minimum; t_½_, elimination half-life; t_max_, time to reach maximum plasma concentration; WT, weight.

^a^Data presented are based on the initial dosing regimen.

**Fig. S1** Mean (SEM) Ratio of Spot UOx:Cr to ULN (**a**) Percent Change From Baseline and (**b**) Actual Values at Each Visit


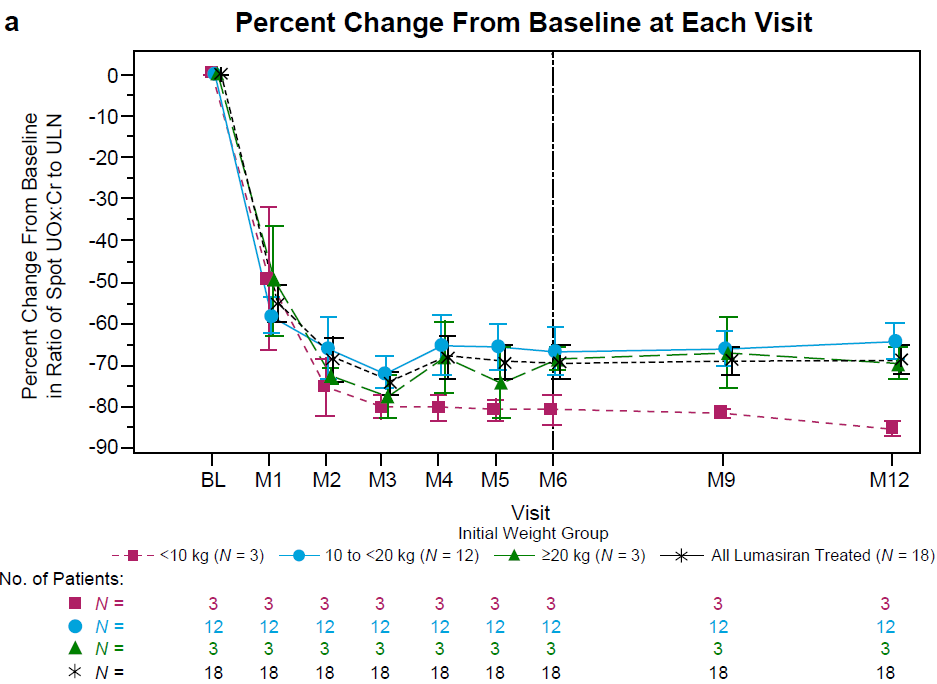


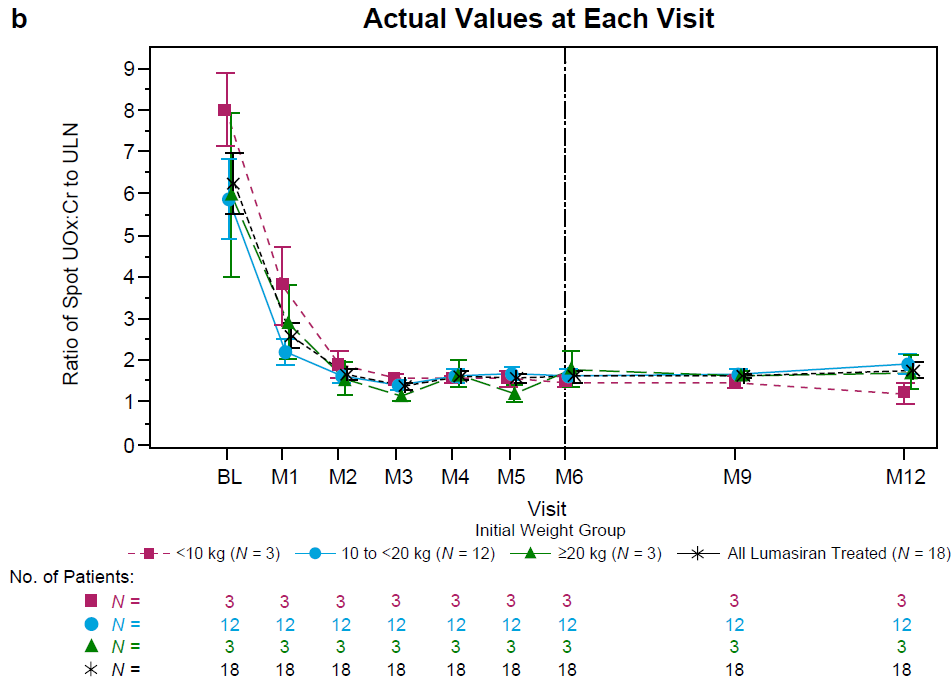


BL, baseline; M, month; ULN, upper limit of normal; UOx:Cr, urinary oxalate:creatinine ratio.

**Fig. S2** Actual eGFR Values in Individual Patients (**a**) <10 kg (*N* = 3), (**b**) 10 to <20 kg (*N* = 12), and (**c**) ≥20 kg (*N* = 3)

Baseline value was the mean of all assessments collected prior to the first dose of lumasiran. eGFR was calculated in patients ≥12 months old at assessment. Two patients did not have eGFR calculated at baseline (age was <12 months).


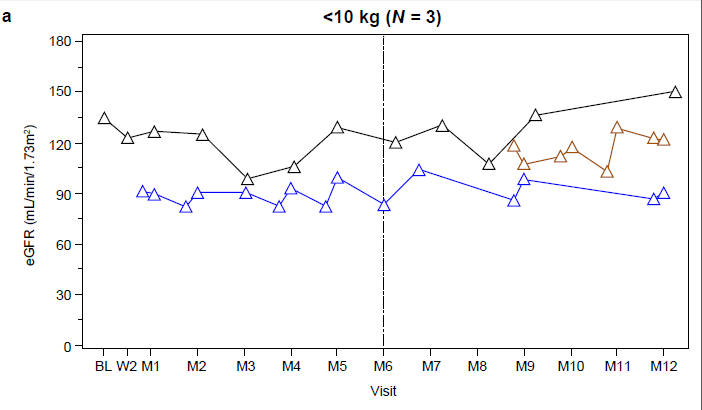


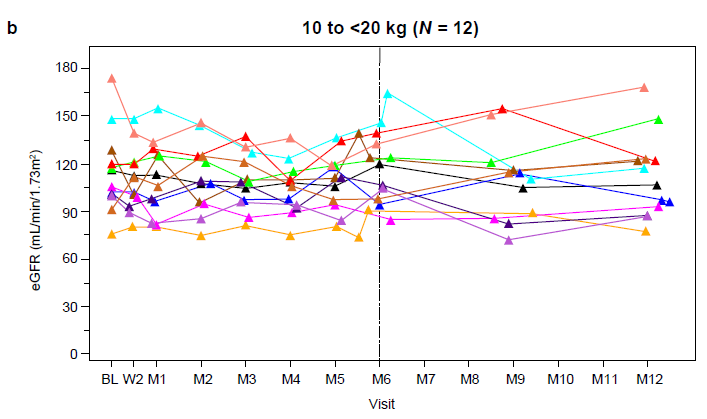


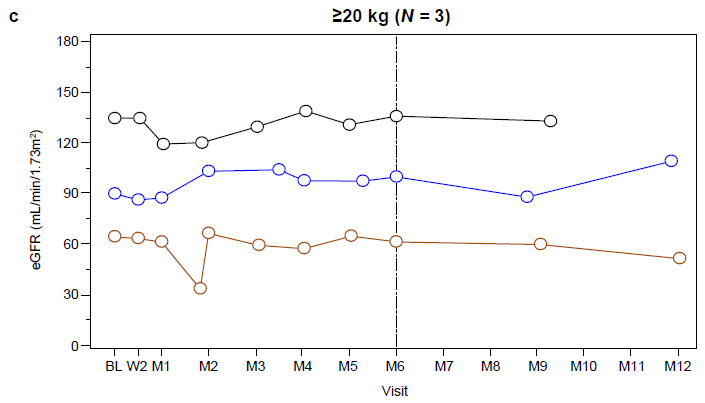
 BL, baseline; eGFR, estimated glomerular filtration rate; M, month; W, week.

**Fig. S3** Mean (SEM) Actual Values in (**a**) Plasma Glycolate and (**b**) Spot Urinary
Glycolate:Creatinine Ratio

Baseline value was the mean of all assessments collected prior to the first dose of lumasiran. For patients in the <10 kg weight subgroup, pharmacodynamic collections were optional at nonquarterly months. In 100 healthy adults, median plasma glycolate was 29.58 µmol/L with a range of 18.14‒54.04 µmol/L.


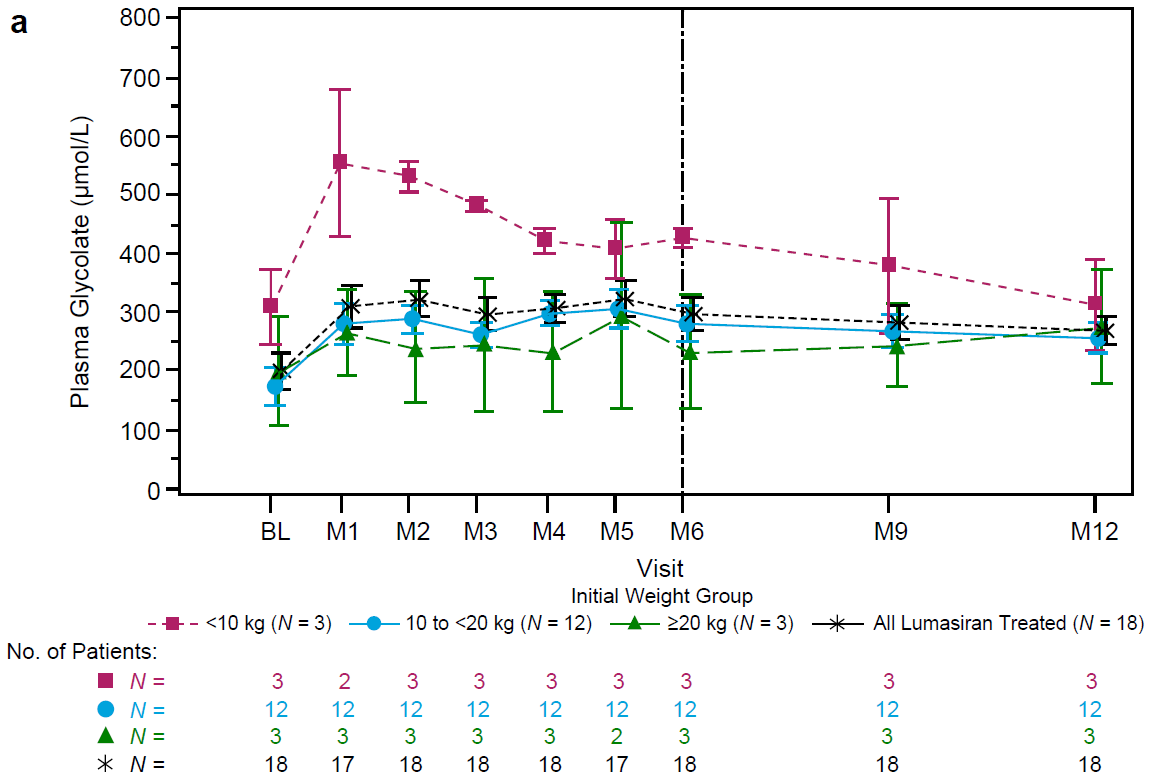


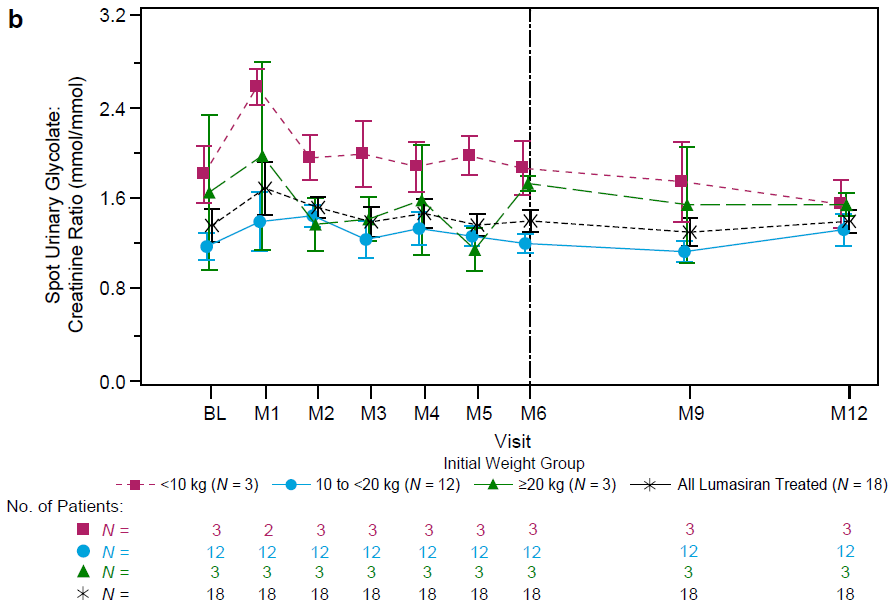


BL, baseline; M, month.
