# Supplementary material for: Efficacy and safety of lumasiran for infants and young children with primary hyperoxaluria type 1: 12-month analysis of the phase 3 ILLUMINATE-B trial
Source: Pediatr Nephrol. 2022 Aug 1;38(4):1075–86. doi: 10.1007/s00467-022-05684-1 (PMC9925547; doi:10.1007/s00467-022-05684-1)
Supplement: Supplementary file 2 — Graphical Abstract (PPTX 99.5 KB) [file 467_2022_5684_MOESM2_ESM.pptx]

## Slide 1
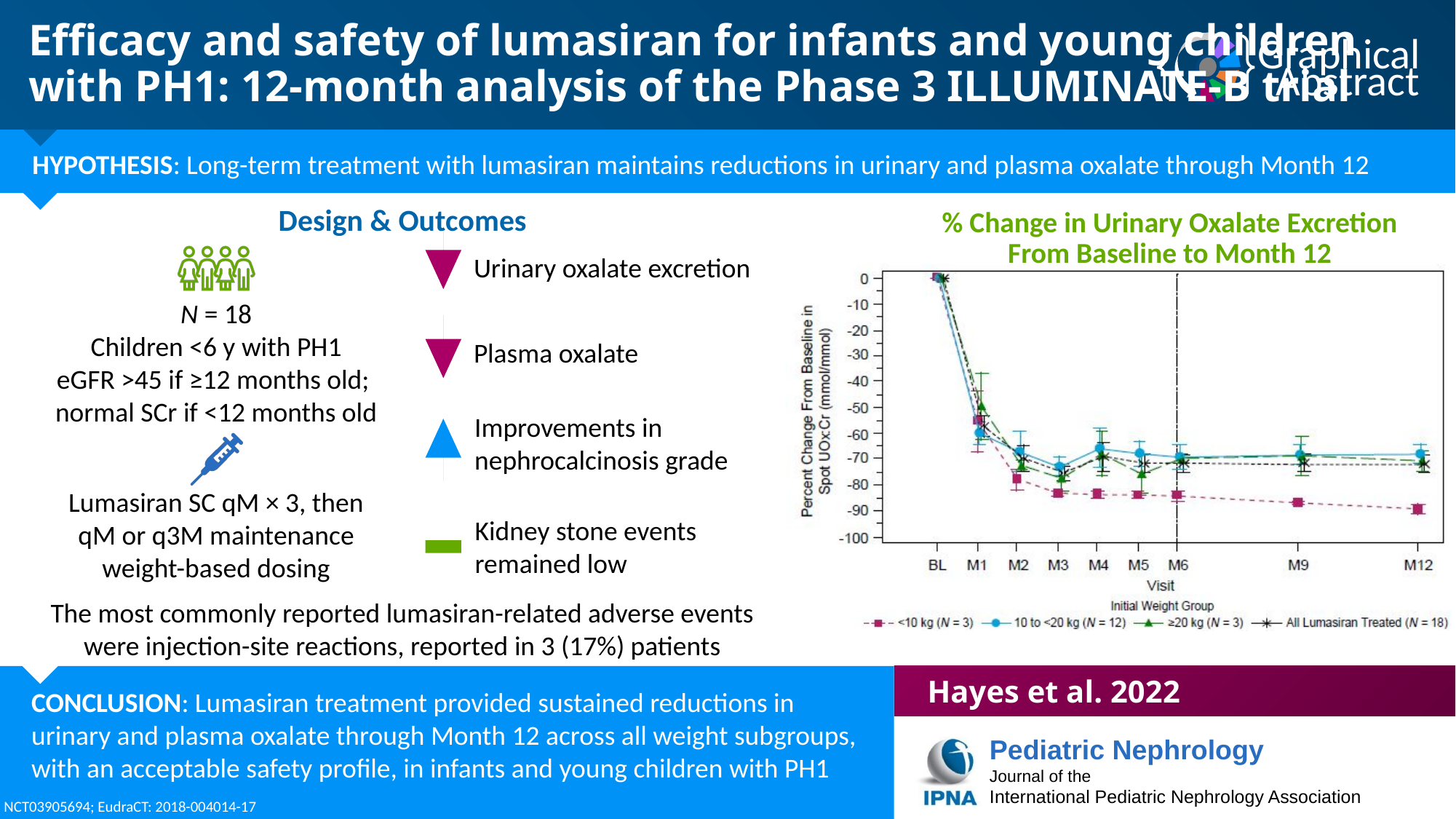

Efficacy and safety of lumasiran for infants and young children
with PH1: 12-month analysis of the Phase 3 ILLUMINATE-B trial
HYPOTHESIS: Long-term treatment with lumasiran maintains reductions in urinary and plasma oxalate through Month 12
Design & Outcomes
% Change in Urinary Oxalate Excretion From Baseline to Month 12
N = 18
Children <6 y with PH1
eGFR >45 if ≥12 months old;
normal SCr if <12 months old
Lumasiran SC qM × 3, then qM or q3M maintenance weight-based dosing
Urinary oxalate excretion
Plasma oxalate
Improvements in nephrocalcinosis grade
Kidney stone events remained low
The most commonly reported lumasiran-related adverse events were injection-site reactions, reported in 3 (17%) patients
Hayes et al. 2022
CONCLUSION: Lumasiran treatment provided sustained reductions in urinary and plasma oxalate through Month 12 across all weight subgroups, with an acceptable safety profile, in infants and young children with PH1
NCT03905694; EudraCT: 2018-004014-17
